# Community-Based Global Health Program for Maltreated Children and Adolescents in Brazil: The Equilibrium Program

**DOI:** 10.3389/fpsyt.2015.00102

**Published:** 2015-07-30

**Authors:** Andrea Horvath Marques, Paula Approbato Oliveira, Luciana Burim Scomparini, Uiara Maria Rêgo e Silva, Angelica Cristine Silva, Victoria Doretto, Mauro Victor de Medeiros Filho, Sandra Scivoletto

**Affiliations:** ^1^Department and Institute of Psychiatry, Medical School, University of São Paulo (USP) – The Equilibrium Program (TEP), São Paulo, Brazil; ^2^Department and Institute of Psychiatry, Medical School, University of São Paulo (USP-PROTOC), São Paulo, Brazil

**Keywords:** traumatized children and adolescents, child neglect, child maltreatment, child abuse, homeless children, mental health, integrated care

## Abstract

The maltreatment of children and adolescents is a global public health problem that affects high- and low-middle income countries (“LMICs”). In the United States, around 1.2 million children suffer from abuse, while in LMICs, such as Brazil, these rates are much higher (an estimated 28 million children). Exposition to early environmental stress has been associated with suboptimal physical and brain development, persistent cognitive impairment, and behavioral problems. Studies have reported that children exposed to maltreatment are at high risk of behavioral problems, learning disabilities, communication and psychiatric disorders, and general clinical conditions, such as obesity and systemic inflammation later in life. The aim of this paper is to describe The Equilibrium Program (“TEP”), a community-based global health program implemented in São Paulo, Brazil to serve traumatized and neglected children and adolescents. We will describe and discuss TEP’s implementation, highlighting its innovation aspects, research projects developed within the program as well as its population profile. Finally, we will discuss TEP’s social impact, challenges, and limitations. The program’s goal is to promote the social and family reintegration of maltreated children and adolescents through an interdisciplinary intervention program that provides multi-dimensional bio-psycho-social treatment integrated with the diverse services needed to meet the unique demands of this population. The program’s cost effectiveness is being evaluated to support the development of more effective treatments and to expand similar programs in other areas of Brazil. Policy makers should encourage early evidence-based interventions for disadvantaged children to promote healthier psychosocial environments and provide them opportunities to become healthy and productive adults. This approach has already shown itself to be a cost-effective strategy to prevent disease and promote health.

## Introduction

Children and adolescent maltreatment is a global public health problem that affects high- and low-middle income countries (“LMICs”) (1–3). Globally, it is estimated that 6% of children under 18 (about 150 million individuals) are victims of maltreatment annually. In urban areas, it is estimated that tens of thousands of children and adolescents are living in poverty, working on the streets, and suffering from abuse and domestic violence (4). In the United States, around 1.2 million children suffer from abuse. These rates are much higher in LMICs, such as Brazil, where an estimated 28 million children suffer from maltreatment (2, 5, 6).

Evidence have shown that children and adolescent exposure to abuse and violence are associated with homelessness, reduced capacity for attachment, increased vulnerability to repeated victimization, suboptimal physical and brain development, and persistent cognitive impairment (7–11). Studies have reported that children exposed to maltreatment are at higher risk of behavioral problems, learning and communication disabilities, internalizing and externalizing psychiatric disorders, and general clinical conditions, such as obesity and systemic inflammation later in life (12, 13). In addition, a growing body of evidence points to the impact of maternal stress during pregnancy on neurodevelopmental disorders (14). Therefore, conditions during pregnancy and early-life can affect adult outcomes and health (15–18).

Conversely, evidence shows that adequate social support is an important protective factor against child maltreatment (19). Enrichment of early childhood environment interventions (e.g., before 5 years of age) for children born in disadvantaged families are associated with better adult social outcomes (e.g., attained higher levels of education, earned higher wages, reduced used of welfare system, and reduced likelihood to commit crime) (20, 21) and better adult health outcomes (fewer behavioral risk factors, such as tobacco, alcohol, and drug use, and better adult physical health) (18). In addition, late-life interventions during puberty (e.g., education) have also shown to promote schooling and improve mental (behavior) and physical health outcome among adults (22). Interestingly, the contribution of early-life factors (family and environment) accounted for at least half of adult health outcomes, independent of the education contribution (18). Early-life interventions through an integrated approach are potentially far more effective in promoting short- and long-term benefits and an overall well-being for the children (18).

In Brazil, community-based services have been established to serve children and adolescents with behavioral and mental problems. Referred to as “Centros de Atenção Psicosocial” or “CAPS” (in English, “Centers for Psychosocial Services”) (23), the program aim to integrate relevant governmental, non-governmental, and community stakeholders (such as advocacy groups and potential clients) to provide active and sustainable dialog, and guide effective interventions tailored to specific populations within a territory (24). However, in practice, the numbers of CAPS available to serve children and adolescents are not enough to address the need (25), and CAPS effectiveness has been limited due to divergent inter-agency agendas and rivalries, and coordination challenges.

To address this gap, a community-based global health program was developed to specifically serve traumatized and neglected children and adolescents in the city of São Paulo, The Equilibrium Program (“TEP”). The aim of this paper is to describe and discuss TEP’s implementation, highlighting its innovation aspects, the research projects developed within the program and its population profile. Finally, we will discuss TEP’s social impact, challenges, and limitations.

## The Equilibrium Program

The Equilibrium Program is a community-based interdisciplinary intervention program that provides multi-dimensional bio-psycho-social treatment integrated with the diverse services needed to meet the unique demands of traumatized and neglected children and adolescents in the city of São Paulo.

The Equilibrium Program is a partnership between faculty at the Department and Institute of Psychiatry of the University of São Paulo Medical School (USP), services providers including health services, social, educational, justice services and child welfare agencies, and several São Paulo municipal departments. For instance, the Department of Health provides financial support for staffing and equipment; the Department of Sports provides physical space (e.g., the program is established inside a Municipal Sports Club), safety, and maintenance. Associated private initiatives have also been developed to support specific interventions, such as communication workshops, dog-assisted therapy, implementing computer systems, and speech therapy. Research funding was provided by grants from the São Paulo Research Foundation (FAPESP), and the University of São Paulo Medical School Foundation (FFM).

The Equilibrium Program was created in response to a shortage of suitable public services available to address the various and broad needs of children and adolescent exposed and addicted to drugs and living on the streets of São Paulo. TEP expands and complements services provided by CAPS and Institutional Refuge Services for Children and Adolescents (Serviços de Acolhimento Institucional Para Crianças e Adolescentes, or “SAICA”), and incorporates innovative aspects, such as multidisciplinary perspectives, networking with health, education, social, and judicial services, all based at a municipal sports club facility.

The Equilibrium Program’s main goal is to provide socio-familiar reintegration for traumatized and neglected children and adolescents with behavioral and mental problems living in foster centers, such as group shelters, or under vulnerable conditions with their families. For children living with their families, the aim is to reinforce family relationships and to provide a safe family environment. Children and Youth Courts are responsible for analyzing and deciding if children are going to be reintegrated with their families or placed in foster and/or adoption centers. Although most children suffer abuse from their own parents, Brazilian law advocates that the family should receive support necessary to remedy the possible causes of domestic violence before placing children for adoption. However, given that most parents suffer from untreated psychiatric illness (26), the improvement of family living conditions and the promotion of parental psychiatric treatment are necessary steps before evaluating the parents’ capacity to properly care for their children. These interventions are provided through social service networks of judicial and child welfare agencies. TEP team supports this process by providing family therapy and reintegration workshops, and by orienting families toward available government benefits.

Group shelters operate under supervision and house about 20 children/adolescents in residences funded by non-governmental organizations and the city of São Paulo. Children and adolescents are referred to TEP by group shelter coordinators, children welfare agencies, and children and juvenile court agencies. More recently, schools and community centers within the vicinity also refer to the program children and adolescents living in vulnerable conditions. An average of 10 new participants is enrolled monthly in TEP.

The first step of the treatment is an initial diagnostic phase, a 4-week assessment performed by a multidisciplinary team (3). Whenever possible, family members are also involved to obtain additional information and participate in the development of a therapeutic plan.

During the diagnostic phase, clinical and psychiatric evaluations are performed to identify psychiatric or medical conditions. In addition, participants are evaluated by neuropsychologists, occupational therapists, art therapists, social workers, educational therapists, and speech therapists to address each participant’s unique problems and identify potential strengths. Psychiatric diagnoses are based on clinical interviews conducted by certified child and adolescent psychiatrists and discussed with a psychiatric coordinator (26).

After the initial assessment, an individualized and integrative therapeutic intervention plan is proposed, which includes periodic psychiatric and pediatric assessment, and individual or group interventions according to each participant’s needs, such as psychotherapy, art and speech therapy, school support, and recreational activities (such as theater, music, and athletics). All activities are integrated within the community center to create a flexible and accepting social environment (27).

A primary case manager is assigned for each participant to ensure coordination and continuity of care between the program activities and external agencies (such as group shelters, schools, and the children and juvenile court systems) and promote school, family, and social reintegration. Weekly treatment team meetings allow adjustments and modification of treatment plans to meet a participant’s evolving needs. Attention to the family’s needs, apart from those of the child or adolescent, is attempted whenever possible. The team thus works actively with other partner organizations to provide continuous care.

A participant’s engagement with the case manager is important to prevent dropouts. Case managers actively maintain contact, especially when participants are absent for more than 2 weeks. Assertive community outreach is often not possible because due to security concerns in some neighborhoods.

Initially, weekly appointments are scheduled. Depending upon a participant’s needs, appointment frequency may increase to three to four times a week, or may be reduced to monthly assessment as participants evolve and are integrated in other care services or programs.

## Innovation

The development of TEP is based on a framework of community-based participatory research in which academic expert partner with community agencies, community stakeholders, and participants to provide assistance in the community programs (28). Development and implementation of TEP considered direct and indirect input from participants, community stakeholders, evidence-based data gathered during assessments and follow-up, as well as national and international service’s experiences and evidence-based practice (3, 23, 29–34). In addition, the program is tailored to the local cultural aspects of São Paulo, the most populous city in the Americas (35), which suffers from high levels urban crime, violence, and organized drug trade (3).

Another of TEP’s innovations is the offering of multidisciplinary services in a safe community setting: a municipal sports club in the vicinity of client residences, and accessible by relevant services (3, 26). The sports club is an attractive environment where children and adolescents are encouraged to participate in healthy activities and develop new abilities. Additionally, a local sports club setting promotes community reintegration and avoids stigmatization – something that often occurs with CAPS users.

One more important innovation is that TEP has adopted a case management system to ensure continuity of care throughout the entire process of treatment and family reintegration. The main activities of the case manager are described in Table 1.

**Table 1:** **Case manager activities**.

No	Case manager activities
1	Lead a multidisciplinary discussion to create an individualized intervention plan
2	Evaluate children status every 3 months applying the Children’s Global Assessment Scale (C-GAS)
3	Implement and follow children’s activities and evaluate children treatment motivation
4	Coordinate with children and adolescent welfare services, juvenile justice system, family members, community support members, NGOs, and private/public organizations
5	Provide educational and vocational support, as well as career guidance
6	Follow-up on dropouts and transfers to other institutions

Another innovative aspect of TEP is the development of research projects to assess population profiles and to evaluate program efficacy. Information on service delivery and client data is systematically collected to improve interventions and support sustainable operations. These data will be essential to promote the program’s future expansion into other areas of Brazil.

Finally, TEP facilitates the development of additional research projects to evaluate the impact of early-life stress on children’s neurodevelopment.

Table 2 describes all the research projects developed by TEP.

**Table 2:** **Main research projects developed by TEP**.

Study	Method	Main results
High rates of psychiatric disorders in a sample of Brazilian children and adolescents living under social vulnerability – urgent public policies implications (36)	351 Children and adolescents underwent a clinical psychiatric evaluation. Demographic and clinical information were obtained by a semi-structured psychiatric interview	Lifetime prevalence of PD was 88.8%, and the most prevalent disorder was substance use (40.4%), mood disorder (35.3%), hyperkinetic (16.2%), and anxiety disorders (8.8%). More than half of subjects had a lifetime history of physical or sexual abuse (58.4%) and 13.1% were both physically and sexually abused. Additionally, many of them suffered from other psychosocial stress, such as admission to a foster center (84.6%) or institutional education (39.8%)
The impact of psychiatric diagnosis on treatment adherence and duration among victimized children and adolescents in São Paulo, Brazil (2)	High lifetime prevalence of psychiatric disorder (86.3%, *n* = 303), such as substance abuse disorder (39%, *n* = 137), mood disorders (37%, *n* = 130), hyperkinetic disorder (16.2%, *n* = 57), and anxiety disorder (8.8%, *n* = 31)	Treatment adherence rate varied with the presence of psychiatric diagnostic: presence of only mood disorders: 79.5%; both mood and substance abuse disorders: 50%; substance abuse disorder alone: 40%; other psychiatric disorder: 75.6%; no psychiatric disorders 72.9%. Living with family was associated with treatment adherence for children with substance abuse disorders. Conversely, it was negatively associated for those with no psychiatric disorders
Intellectual deficits in Brazilian victimized children and adolescents: A psychosocial problem? (37)	150 Children and adolescents underwent neuropsychological assessment of estimated intellectual quotient (EIQ). Maltreatment history was assessed by reviewing medical records to identify diagnoses related to socioeconomic and psychosocial circumstances	Average IQ was 87.25 (lower-average; SD 15.54), and a large number of patients (25.3%; *N* = 38) with below-average scores, contrary to what was expected (8.9%)
Auditory processing in children and adolescents in situations of risk and vulnerability (38)	Auditory processing tests were applied to a group of 27 individuals (11 children between 7 and 10 years old and 16 adolescents between 11 and 16 years old), of both sexes, in situations of social vulnerability, compared with an age-matched control group of 10 children and 11 adolescents without complaints. The BAEP test was also applied to investigate the integrity of the auditory pathway	Participants with social vulnerability had significantly poorer performance in the behavioral auditory processing tests, despite their unaltered auditory brainstem pathways, as shown by their normal results in the BAEP test
Association of child maltreatment and psychiatric diagnosis in Brazilian children and adolescents (39)	351 Patients (mean age of 12.47), 68.7% male, and 82.1% underwent psychiatric evaluations based on the Kiddie-Sads-Present and Lifetime Version (K-SADS). Two different methods were used to evaluate maltreatment: medical records were reviewed to identify previous diagnoses related to socioeconomic and psychosocial circumstances, and the Childhood Trauma Questionnaire (CTQ) was used to obtain a structured history of trauma	The most frequent psychiatric diagnoses were substance use disorders, affective disorders, and specific disorders of early childhood, whereas 13.67% had no psychiatric diagnosis. All patients suffered neglect, and 58.4% experienced physical or sexual abuse. History of multiple traumas was only associated with a diagnosis of substance use disorder. Mental retardation showed a strong positive association with physical abuse and emotional neglect. However, negative correlation was found presence of a history of multiple traumas and mental retardation
Neuropsychological and psychiatric profile of adolescents exposed to maltreatment (40)	108 Adolescents were classified according to the scores in CTQ: GMT1 Group (Mild Maltreatment, *n* = 35), GMT2 (group of moderate to severe maltreatment, *n* = 19) and CG (comparison group, *n* = 54). Neuropsychological evaluation was performed, focusing on visual perception and attention spam (first functional unit), processing and retention of information (second functional unit) and executive functioning (third functional unit). Scales were applied for evaluation of impulsivity, hyperactivity, attention, and opposition symptoms (SNAP-IV, BIS-11)	Maltreatment groups had a worse intellectual functioning compared to GC, while the worst performance was found in GMT2 (*p* < 0.001). Lower IQ measures were associated to impairment on the three functional units and to more symptoms of inattention and hyperactivity. Worse performance on tests for evaluation of the second functional unit was observed on GMTs groups. Negative correlation was observed between CTQ scores and performance in all functional units. However, higher CTQ score were positively correlated with impulsivity, opposition, isolated symptoms of inattention, and mixed symptoms of inattention and hyperactivity
Inter-hemispheric transfer of information deficits and white matter integrity in the corpus callosum in adolescents with a history of severe maltreatment: a DTI and neuropsychological study (41)	41 Maltreated adolescents (MAL) and 39 controls (CON) underwent diffusion tensor imaging (DTI) and neuropsychological assessment	A trend toward a reduced fractional anisotropy (FA) in the corpus callosum was observed on MAL (0.05 < *p* < 0.10). MAL had poorer neuropsychological performance on the following tests: Wisconsin card sorting test (WCST) and crossed finger localization test (CFLT). The deficits found in WCST and CFLT could be associated with corpus callosum abnormalities in maltreated adolescents
Cognitive performance, history of maltreatment, and psychiatric symptoms in mothers of children and adolescents who are victims of maltreatment (42)	29 Mothers of children and adolescents served by The Equilibrium Program underwent psychological assessment through the Mini International Neuropsychiatric Interview, cognitive assessment through vocabulary subtests and cubes on the Wechsler adult intelligence scale-III (WAIS III). Additionally, a historical assessment of trauma and interview for social characterization were performed	The majority of mothers did not complete elementary education, and earned less than half of the minimum wage. It was determined that the IQ of at least half of the sample (*n* = 15, 51.7%) was below the median, which indicated compromised cognitive development. Results also indicated an occurrence of all types of maltreatment, such as physical, sexual, and emotional abuse and physical and emotional negligence. A large part of the sample demonstrated criteria for major depression (*n* = 19, 65.5%), post-traumatic stress syndrome (*n* = 10, 34.5%), and general anxiety disease (*n* = 9, 31%)
Functional outcomes of maltreated children and adolescents in a community-based Rehabilitation program in Brazil: 6-month improvement and baseline predictors (43)	This study sought to implement outcomes monitoring and to review outcome data from TEP. From 452 maltreated children and adolescent, about half (*n* = 230) of the participants were successfully evaluated using the Children’s global assessment scale (C-GAS) at entry, 3, and/or 6 months later. Analysis of outcomes used hierarchical linear modeling of functional change from baseline	With a baseline C-GAS score of 51.7 (SD = 14.22), average improvement was 2.8 and 5.5 points at 3 and 6 months, respectively (reflecting small to moderate effect sizes = 0.20 and 0.39). Improvement was associated with problems related to upbringing (*p* < 0.02) at entry and absence of Physical abuse (*p* < 0.05) and Negative life events in childhood (*p* < 0.05) but was not associated with sociodemographics or any specific psychiatric diagnosis. This study showed that outcomes monitoring is feasible in a community-based program in a developing country
Decrease in salivary cortisol levels in adolescents victims of sexual abuse (44)	77 Adolescents were enrolled to evaluated trauma (CTQ) and cortisol levels (2 times- fasting and after CTQ questionnaire application). The Estimated Intelligence Quotient (IQ) was obtained through the Vocabulary and Block Design subtests on the WISC-III. Associations between previous instances of trauma and cortisol levels were performed through simple linear correlation test	The average IQ of was 110.46 (SD = ± 14.03, average IQ). Lower levels of salivary cortisol were found in victims of sexual abuse after evoking memories of traumatic events (ρ = -0.237, β = -0.006, *p* = 0.04), in comparison with the rest of the sample, which had been exposed to other types of trauma. Such changes could be related to the body’s neuro-adaptive mechanisms to stressful situations, with a likely negative impact on the brain’s maturation process during adolescence
Auditory-perception analysis of voice in maltreated children and adolescents (45)	136 Children and adolescents (average age 10.2 years, 78 male) were assessed. Speech evaluation was performed (involving the aspects of oral and written communication, as well as perceptual analysis-voice hearing, made through the GRBASI scale). Psychiatric diagnosis was performed to the ICD-10 diagnostic criteria and by the application of K-SADS; the global functioning was evaluated by means of the C-GAS scale	The prevalence of vocal change was 67.6% (79.3% aged up to 12 years, 56.5 of male), without statistic differences for age and gender. Of patients with vocal change, 92.3% presented other disorders of communication, significantly associated with certain disorders. There was no association between vocal changes and psychiatric diagnosis. The voice change was associated with a loss of 7 points in the global functioning
Co-occurrence of communication disorder and psychiatric disorders on maltreated children and adolescents: relationship with global functioning (Stivanin et al., submitted)	143 Maltreated children and adolescents (55.8% male) were enrolled and underwent clinical communication, psychiatric evaluations, and global functioning assessment by applying Children’s global assessment scale (C-GAS)	Four groups emerged by evaluation: Group 1 (4.9%) no psychiatric disorders; Group 2 (18.2%) presence of PD; Group 3 (23.8%) presence of CD; Group 4 (*n* = 76, 53.1%) presence of both PD and CD. Significant differences on C-GAS were observed between groups. There was a high prevalence of PD and CD in maltreated population. The presence of PD has a major impact on C-GAS, and the simultaneous presence of CD increased the already impaired function of PD
Inattention, hyperactivity, and opposition associated with maltreatment and psychosocial conditions: the role of environment on ADHD symptomatology (Oliveria et al., submitted)	108 Adolescents were evaluated by K-SADS-PL, SNAP-IV, and CTQ. They were classified into three groups: mild maltreatment (*n* = 35), moderate to severe maltreatment (*n* = 19), and comparison group (*n* = 54). When comparing the groups, psychosocial and IQ variables were controlled	Inattention symptoms were presented in 42.1% of moderate to severe maltreatment group and 3.7% on comparison group. Positive correlation was observed between CTQ total score and inattention, combined type (inattentive/hyperactive), and opposition symptoms. Intellectual quotient was associated with ADHD-inattentive subtype and ADHD-combined type. Educational level and institutional care were associated with ADHD hyperactive subtype

*PD, psychiatric disorders; IQ, intellectual quotient; BAEP, brainstem auditory evoked potential; K-SADS, Kiddie-Sads-Present and Lifetime Version; CTQ, childhood trauma questionnaire; GMT1, group mild treatment; GMT2, group of moderate to severe treatment; CG, comparison group; SNAP-IV, Swanson, Nolan and Pelham Questionnaire; BIS, Barratt impulsiveness scale; MAL, maltreated adolescents; CON, controls; FA, fractional anisotropy; WCST, Wisconsin card sorting test; CFLT, crossed finger localization test; WAIS III, Wechsler adult intelligence scale-III; C-GAS, assessment scale; GRBASI, perceptual-auditory voice assessment; PD, psychiatric disorders; CD, conduct disorder; ADHA, attention deficit hyperactivity disorder; K-SADS_PL, Kiddie-Sads-Present and Lifetime Version*.

## Population Profile

Studies of patients served by TEP have identified the prevalence of psychiatric diagnoses and an association with neuropsychological deficits, treatment adherence, and clinical evolution (Table 2). These studies demonstrated the feasibility of monitoring functional outcomes for TEP patients as an example of data monitoring for community-based programs for maltreated children in LMICs (3, 43).

In comparison with national epidemiological data (46), the lifetime prevalence of psychiatric disorder were higher in this population [e.g., substance abuse disorder (39–31.9%), mood disorders (37–33.8%), hyperkinetic disorder (16.2–20.8%), conduct disorder (15.9%), anxiety disorder (11.7–8.8%), and developmental disorder (5.7%)] and have shown to impact treatment adherence (2, 39, 43) (see Table 2).

Treatment adherence was associated with slightly higher functioning (C-GAS Score β = 0.02, *p* < 0.01), and was less likely to have a history of physical abuse (β = 46, *p* < 0.03) (43). In another study, treatment adherence was better for those with only mood disorders (79.5%) than for those with substance abuse disorder (40%) or without any psychiatric disorders (72.9%) (2).

Most common social problems reported were negative life events, such as loss of a love relationship, removal from the home, experiencing an altered pattern of family relationships in childhood (95.3%); problems related to upbringing (84.1%), a family history of mental illness (57.5%), physical abuse (32.5%), sexual abuse (15.7%), and criminal involvement (11.5%) (43). These data emphasize the importance of global psychiatric assessment. Paying attention only to substance use disorders can lead to underestimation of other mental health problems (36). Therefore, continuous efforts are needed to improve psychiatric assessment, and distinct approaches, like dimensional and categorical measures, should be considered (47).

Improvement was observed after 3 and 6 months of follow-up (small to moderate effect sizes, 0.20 and 0.39) and was positively associated with problems related to upbringing, negatively associated with physical abuse and negative life events in childhood, and not associated with any socio-demographic characteristic or psychiatric diagnosis (43). However, some limitations have to be address, such as high rates of dropouts and lack of control group. Only half of the subjects had completed the treatment plan, which likely reflects the challenge of adherence in the program. Although there was a lack of a control group and no specific evidence-based treatments were applied, it is notable and encouraging that making services available from a multidisciplinary team in a safe supportive environment was associated with significant improvement within only 6 months period, albeit with a small to moderate effect size (43).

Other studies have also shown the impact of early life stress on children’s cognitive function, revealing a negative impact on intellectual functioning (43), poor neuropsychological performance associated with corpus callosum abnormalities (37, 48, 49), poor performance in behavioral auditory processing tests (38), deficit in auditory perception (48), and higher prevalence of communication disorder associated with psychiatric diagnosis (50) (Table 2).

Finally, another important data collected during the first year of the program is related to family’s member history. Child victims of abuse often lived in dysfunctional families, with a high frequency of untreated psychiatric problems (36). In addition, family members who reported involvement with alcohol and drugs also reported being abused as children and often did not receive any treatment (26). These findings corroborate with previous studies that report the association between children maltreatment and increased risk of substance use and other psychopathology in adulthood (51). These factors hinder the promotion of a healthy family environment. Therefore, successful family reintegration should address the cycle of unfavorable environment – psychic disintegration – violence, and promote parental mental and physical health (52).

## Social Impact

The Equilibrium Program has demonstrated itself as a feasible program promoting important positive impacts on children and adolescents outcomes. Data collected from September 2007 through December 2014 are summarized below.

- 92,111 appointments
- Average of 1,046 appointments monthly
- 56.4% (344) clients on treatment or that completed the treatment plan (107–31.1% referred to other treatment centers)
- 47.1% (287) children/adolescents were reintegrated into families (original or step families)
- 42.8% (261) dropout
- 6.3% (39) released due to relocation with their families outside the city
- 0.65% (4) admitted into witness protection programs
- 0.16% (1) deaths
- 1,196 caregiver supervision sessions held from November 2011 to September 2013.

## Challenges and Limitations

Challenges observed during TEP’s development and implementation provided relevant information to improve and expand similar efforts in Brazil and in other LMICs. Several challenges and limitations were observed along the 7 years of TEP’s activity.

First was the creation of a multidisciplinary service in a safe and non-stigmatized setting in the vicinity of user residences and safely accessible to providers. Second was the development and maintenance of the partnership between a university, other service providers (such as social services, school, health provider, and child welfare agencies) and the municipal government to address the needs of the population and to provide long-term financial support for the program. Another challenge was the monitoring of program outcomes. Although the program operates in a real-world setting, associated research projects are required to guide effective interventions, evaluate population profiles, and collect data to support of the development of public policies targeted to this specific population. This aspect was overcome by creating collaboration with national international academic experts, and pursuing grants from Brazilian funding agencies.

Another major challenge and limitation were the evaluation of TEP’s impact on the community. Although TEP could have potentially exerted a positive impact on the neighboring environment and decreased violence rates, these data were not collected due to lack of financial support. Finally, after few years of operation, the team learned that devoting attention and support to professionals working with children and adolescents, especially caregivers, is as important as addressing their needs. Therefore, providing support to reduce caregivers’ work stress, improving stability, and providing a suitable environment in group shelters is an essential strategy that can contribute to children’s outcome.

## Conclusion

Children and adolescent maltreatment is a global public health problem affecting high-income and LMICs. Studies have shown that children exposed to maltreatment are at high risk of learning and communication disabilities, persistent cognitive impairment, behavioral problems, psychiatric disorders, and general clinical conditions, such as obesity and systemic inflammation later in life (13).

A better understanding of the multiple factors affecting mental health status of children and adolescents is necessary but not sufficient for the development of novel treatment approaches required to serve this population (53). Services models to treat traumatized children applied in developed countries, such as assertive community approaches, may not be compatible in deeply impoverished urban neighborhoods in LMICs due to the high levels of urban crime, violence, and the organized drug trade (30, 31).

In Brazil, services available to homeless children or in supervised group shelters are frequently inadequate, fragmented, and poorly coordinated (32). There is little interaction between social and health systems, social support is precarious, and health care follow-up is impeded by frequent changes of client residence and the impracticability of assertive community outreach due to a lack of safety for community workers (54). As a result, it is difficult to maintain the bonds that sustain the confidence and trust of children and adolescents in these situations. Long-term follow-up, from the street to the point of family reintegration, is essential to countering further family disaffection.

The Equilibrium Program was developed to fill this gap. TEP is an interdisciplinary intervention program that provides multi-dimensional bio-psycho-social treatment for multiply traumatized children and adolescents, integrating widely diverse services needed to meet the unique demands of this population (e.g., general health care assistance, schools, social services, child welfare programs, and the criminal justice system) in a safe, accessible setting. The program’s main goal is to promote social and family reintegration of maltreated children and adolescents. TEP was developed and implemented through a partnership between academic psychiatrists from the University of São Paulo Medical School, the São Paulo municipal government, and potential users. TEP innovative aspects are as follows: (a) program development was guided by principles emphasizing acceptability to consumers, flexibility in addressing diverse client needs, and placing a focus on high-risk sub-populations within a supportive environment; (b) TEP is located in a community sports center near to many of the shelters close to downtown São Paulo. The center is open to the local community, serving to facilitate the social reintegration and stigma reduction process among those children and their families in a safe and secure environment; (c) TEP offers comprehensive mental and physical health care along with social services, specialized services, and support for school attendance while participating in social and recreational activities with their peers; (d) TEP is also performing research projects to assess population profiles and program evaluation efficacy by monitoring program outcomes as it operates in real-world setting to guide effective interventions.

After 7 years of activity and based on research developed in a real-world setting to monitor program efficacy, data show that TEP is a feasible and sustainable program that helps to revert the inter-generational violence cycle through a multidisciplinary work in a non-stigmatized environment, which emphasizes building connections between users, staff, and all stakeholders.

Box 1 **Key Features of TEP.**

(1) Offers intensive professional services that are accessibly located within the community in a context primarily associated with recreational activities,
(2) Offers an environment far away from adverse environmental elements in the community,
(3) Is accessible to majority of service providers located elsewhere in the city, and
(4) Promotes active community and family reintegration of maltreated children and adolescents.

## Conflict of Interest Statement

The authors declare that the research was conducted in the absence of any commercial or financial relationships that could be construed as a potential conflict of interest.
